# α/β Hydrolase Domain-containing 6 (ABHD6) Degrades the Late Endosomal/Lysosomal Lipid Bis(monoacylglycero)phosphate*

**DOI:** 10.1074/jbc.M115.669168

**Published:** 2015-10-21

**Authors:** Maria A. Pribasnig, Irina Mrak, Gernot F. Grabner, Ulrike Taschler, Oskar Knittelfelder, Barbara Scherz, Thomas O. Eichmann, Christoph Heier, Lukas Grumet, Jakob Kowaliuk, Matthias Romauch, Stefan Holler, Felix Anderl, Heimo Wolinski, Achim Lass, Rolf Breinbauer, Gunther Marsche, J. Mark Brown, Robert Zimmermann

**Affiliations:** From the ‡ Institute of Molecular Biosciences, University of Graz, 8010 Graz, Austria,; the § University of Technology, 8010 Graz, Austria,; the ¶ Institute of Organic Chemistry, Medical University of Graz, 8010 Graz, Austria, and; the ‖ Institute of Experimental and Clinical Pharmacology, Department of Cellular and Molecular Medicine, Cleveland Clinic Lerner Research Institute, Cleveland, Ohio 44195

**Keywords:** endocannabinoid, endosome, lipid metabolism, lysosome, phospholipase, bis(monoacylglycero)phosphate, lysobisphosphatidic acid

## Abstract

α/β Hydrolase domain-containing 6 (ABHD6) can act as monoacylglycerol hydrolase and is believed to play a role in endocannabinoid signaling as well as in the pathogenesis of obesity and liver steatosis. However, the mechanistic link between gene function and disease is incompletely understood. Here we aimed to further characterize the role of ABHD6 in lipid metabolism. We show that mouse and human ABHD6 degrade bis(monoacylglycero)phosphate (BMP) with high specific activity. BMP, also known as lysobisphosphatidic acid, is enriched in late endosomes/lysosomes, where it plays a key role in the formation of intraluminal vesicles and in lipid sorting. Up to now, little has been known about the catabolism of this lipid. Our data demonstrate that ABHD6 is responsible for ∼90% of the BMP hydrolase activity detected in the liver and that knockdown of ABHD6 increases hepatic BMP levels. Tissue fractionation and live-cell imaging experiments revealed that ABHD6 co-localizes with late endosomes/lysosomes. The enzyme is active at cytosolic pH and lacks acid hydrolase activity, implying that it degrades BMP exported from acidic organelles or *de novo*-formed BMP. In conclusion, our data suggest that ABHD6 controls BMP catabolism and is therefore part of the late endosomal/lysosomal lipid-sorting machinery.

## Teaser

**Background:** Bis(monoacylglycero)phosphate (BMP) plays a key role in the degradation and sorting of lipids in acidic organelles.

**Results:** ABHD6 degrades BMP and co-localizes with late endosomes/lysosomes. Knockdown of ABHD6 increases hepatic BMP levels.

**Significance:** ABHD6 is part of the late endosomal/lysosomal lipid-sorting machinery.

## Introduction

α/β Hydrolase domain-containing 6 (ABHD6) was originally identified as brain monoacylglycerol (MG)^3^ lipase, capable of degrading the endocannabinoid 2-arachidonoylglycerol (2-AG)(1). This MG species acts as an endogenous activator of cannabinoid receptors, and its biological effects are mimicked by Δ^9^-tetrahydrocannabinol, the major psychoactive component of marijuana (2). Further studies have revealed that ABHD6 is differentially expressed in cancer cell lines and highly present in Ewing tumors, indicating that it might be an interesting diagnostic or therapeutic target (3, 4). Marrs *et al.* (5) have reported that ABHD6 controls the accumulation and efficacy of 2-AG at cannabinoid receptors. This finding was surprising because most of the 2-AG-degrading activity detected in brain preparations was ascribed to monoglyceride lipase, and monoglyceride lipase-deficient mice exhibit a strong increase in 2-AG levels (10- to 50-fold) (6–8). In comparison, ABHD6 inhibition only causes minor changes in 2-AG levels. However, this moderate increase in 2-AG induces the activation of cannabinoid receptor, type 1 (5), and recent evidence suggests that pharmacological ABHD6 inhibition has neuroprotective effects in a mouse model of traumatic brain injury (9). Similarly, neuroprotective effects have been observed in response to inhibition of the endocannabinoid-degrading enzymes monoglyceride lipase and fatty acid amid hydrolase (10, 11). Furthermore, it has been demonstrated that ABHD6 blockade exerts antiepileptic effects in a genetic mouse model of juvenile Huntington disease known to exhibit dysregulated endocannabinoid signaling (12). It is believed that ABHD6 is active in specific cells or subcellular compartments and responsible for the fine-tuned modulation of 2-AG levels (13).

Very recent evidence implies that ABHD6 plays an important role in the development of obesity and liver steatosis. Selective knockdown in liver and adipose tissue or pharmacological inhibition of ABHD6 protects mice from high-fat diet-induced obesity and hepatic steatosis (14). Furthermore, ABHD6 acts as a negative modulator of glucose-stimulated insulin secretion (15). Mechanistically, it has been proposed that the lack of ABHD6 leads to increased levels of long-chain saturated MGs that bind and activate the vesicle priming protein Munc13-1, thereby inducing insulin exocytosis (15). However, ABHD6 is not a selective MG hydrolase (14) and, therefore, may affect lipid signaling and metabolism by other pathways. In addition to MG, ABHD6 is capable of degrading acidic lysophosphospholipids and affects glycerophospholipid metabolism (14).

Here we aimed to further explore the function of ABHD6 in lipid catabolism. Our data demonstrate that both mouse and human ABHD6 degrade bis(monoacylglycero)phosphate (BMP), also known as lysobisphosphatidic acid, with high specific activity. This lipid is enriched in intraluminal vesicles (ILVs) of late endosomes (LE), where it represents ∼15% of total phospholipids (16). BMP favors the formation of ILVs in LE and their back fusion with the limiting membrane and plays a central role in lipid digestion and sorting (17–19). Our observations suggest that ABHD6 controls BMP hydrolysis and is therefore part of the late endosomal/lysosomal lipid sorting machinery.

## Experimental Procedures

### 

#### 

##### Materials

The following lipids were purchased from Avanti Polar Lipids: dioleoyl-phosphatidic acid, dioleoyl-phosphatidylcholine, dioleoyl-phosphatidylethanolamine, phosphatidylglycerol (PG), dioleoyl-phosphatidylinositol, and dioleoyl-phosphatidylserine and the corresponding lysophospholipids monooleoyl(*sn-1*)-lysophosphatidic acid, -lysophosphatidylcholine, -lysophosphatidylethanolamine, -lysophosphatidylglycerol, -lysophosphatidylinositol, and -lysophosphatidylserine; dioctanoyl-phosphatidylcholine and dioctanoyl-PG; bis-dioleoyl cardiolipin; bis(dioleoylglycero)phosphate; *N*-acylphosphatidylethanolamine; plasmanylcholine; *sn-3*,*3*′-oleoyl-BMP(*S*,*S* and *R*,*R*); 1-oleoyl-*N*-heptadecanoyl-d-*erythro*-sphingosine; and 1,2-diacyl-3-*O*-β-d-galactosyl-*sn*-glycerol. The following lipids were purchased from Sigma-Aldrich: dioleoylglycerol, trioleoylglycerol, glyceryl trioctanoate, monoolein, retinyl palmitate, arachidyl laurate, cholesteryloleate, and oleoyl-carmitin. Methylpalmitate, ethylpalmitate, propylpalmitate, and butylpalmitate as well as *sn-2*,*2*′-oleoyl-BMP(*S*,*S*) were synthesized according to published procedures (20, 21). Labeled BMP was synthesized using oleic acid ^13^C_18_ (Sigma-Aldrich) according to Refs. 21.

##### Cloning

Mouse and human ABHD6 (NP_079617 and NP_065727, respectively) were amplified from cDNA using the following primers: ABHD6, 5′-GCA TAT GAA TTC GAT CTC GAT GTG GTT AAC ATG TT-3′ (forward) and 5′-GCT AAT CTC GAG TCA GTT CAG CTT CTT GTT GTC TG-3′ (reverse); hABHD6, 5′-AAG GAT CCA TGG ATC TTG ATG TGG TTA AC-3′ (forward) and 5′-AAC TCG AGT CAG TCC AGC TTC TTG TTG-3′ (reverse).

PCR products were ligated to compatible restriction sites of the eukaryotic expression vector pcDNA4/HisMaxC (Invitrogen). A control pcDNA4/HisMax vector expressing β-galactosidase was provided by the manufacturer (Invitrogen). Point mutations were introduced by using the GeneTailor site-directed mutagenesis system (Invitrogen) according to the instructions of the manufacturer. For live-cell imaging experiments, the coding sequence (without stop codon) of murine ABHD6 was subcloned into the pECFP-N1 vector (Clontech Laboratories, Inc., Mountain View, CA) using the XhoI and SacII restriction sites. For the expression of tagless ABHD6, the coding sequence was subcloned into pECFP-N1, including the stop codon.

##### Expression of Recombinant Proteins in Yeast and COS-7 Cells

GST-tagged murine ABHD6 was expressed in *Saccharomyces cerevisiae* and purified by affinity chromatography as described previously (14). The purified enzyme was stored in buffer containing 50 mm Tris/HCl (pH 8), 0.25 m sucrose, 1 mm EDTA, and 0.1% Nonidet P-40 at −80 °C. COS-7 cells (SV-40 transformed monkey embryonic kidney cells; ATCC, catalog no. CRL-1651) were cultivated in DMEM (Gibco, Invitrogen) containing 10% FCS (Sigma-Aldrich) and antibiotics (penicillin (100 IU/ml) and streptomycin (100 μg/ml)) under standard conditions (95% humidified atmosphere, 37 °C, 5% CO_2_). Cells were transfected with recombinant DNA complexed to Metafectene (Biontex Laboratories GmbH) in FCS-free medium. After 4 h, the medium was changed to serum-containing medium, and cells were harvested 48 h post-transfection.

##### Determination of BMP Hydrolase (BMPH) Activity

For determination of enzyme activity in COS-7 cell lysates, cells overexpressing recombinant proteins were washed with ice-cold PBS and disrupted by brief sonication in homogenization buffer (0.25 m sucrose, 1 mm EDTA, 1 mm Tris/HCl (pH 8), 150 mm NaCl, 20 μg/ml leupeptin, 2 μg/ml antipain, and 1 μg/ml pepstatin). Lysates were centrifuged at 1000 × *g* for 20 min at 4 °C, and the supernatant was used for measuring enzyme activity. Tissue BMPH activity was detected in overnight-fasted wild-type mice. For that purpose, tissues were washed with ice-cold PBS and homogenized on ice in extraction buffer (0.25 m sucrose, 1 mm EDTA, 1 mm Tris/HCl (pH 8), 50 mm NaCl, 0.1% Nonidet P-40, 20 μg/ml leupeptin, 2 μg/ml antipain, and 1 μg/ml pepstatin) using an Ultra Turrax (IKA, Staufen, Germany). Lysates were centrifuged at 10,000 × *g* for 20 min at 4 °C. Floating lipid droplets accumulating at the top of lysates were removed, and the remaining supernatant was used to measure enzyme activity. For determination of BMPH activity in lysosomes, the lysosomal fraction (LF), obtained by differential centrifugation (see below), was resuspended in extraction puffer.

Lipid substrates were prepared by dispersing BMP and other lipids in substrate buffer containing 50 mm Tris/HCl (pH 8), 100 mm NaCl, 1 mm EDTA, and 5 mm CHAPS by brief sonication. Activity assays were typically performed at pH 8 and in the presence of 2 mm lipid substrate unless indicated otherwise. Purified ABHD6 or cell/tissue preparations (10 μl) were incubated with 40 μl of lipid substrate at 37 °C for the indicated time periods. Thereafter, the reaction was terminated by addition of 0.25 ml of chloroform/methanol (2:1) containing 1% acetic acid. After vortexing, the organic phase containing free fatty acids (FFA) was dried using a SpeedVac concentrator, and the lipid pellet was redissolved in 1% Triton X-100. FFA release was quantified using a commercially available kit (NEFA C kit, Wako Chemicals). FFA detected in lysates incubated under identical conditions but in the absence of BMP served as a blank (∼10% and 5% of total activity in brain lysates and COS-7 lysates). BMPH activity in liver lysates was determined using a substrate containing ^13^C-labeled oleic acid in the *sn-2* positions of BMP(*S*,*S*) because of high endogenous FFA levels. The release of ^13^C-labeled oleic acid was quantified after fatty acid derivatization with *N*-(4-aminomethylphenyl)pyridinium (Cayman Chemicals) by LC-ESI-MS.

##### Tissue Fractionation

For differential centrifugation, livers of overnight-fasted C57Bl/6 mice were minced in ice-cold buffer A (1 g of tissue/4 ml of buffer containing 0.25 m sucrose, 10 mm Tris/HCl (pH 7.4), 20 mg/ml leupeptin, 2 mg/ml antipain, and 1 mg/ml pepstatin) and disrupted using a precooled Potter-Elvehjem (6 strokes at 600 rpm). The homogenate was centrifuged at 1000 × *g*, 4 °C for 10 min to remove cell debris and nuclei. Thereafter, the 1000 × *g* supernatant was centrifuged at 12,000 × *g* for 10 min to yield the 12,000 × *g* supernatant (crude lysosomal fraction). To induce mitochondrial swelling, CaCl_2_ was added at a final concentration of 8 mm to the crude lysosomal fraction, and samples were incubated at 4 °C for 15 min. Then samples were centrifuged at 25,000 × *g* for 15 min at 4 °C. The pellet was suspended in 10 mm Tris/HCl buffer (pH 7.4) and 150 mm KCl and resedimented at 25,000 × *g* for 15 min at 4 °C to obtain the LF. Samples of each fraction (10 μg of total protein) were subjected to Western blot analysis and analyzed for organelle markers and ABHD6.

For the preparation of a discontinuous gradient, the 1000 × *g* supernatant of liver homogenates was adjusted to 30% iodixanol (Optiprep^TM^). The sample was applied to the bottom of a prechilled ultracentrifuge tube and overlaid with decreasing concentrations of iodixanol solution in buffer A (20%, 15%, 10%, and 0% iodixanol). Subsequently, samples were subjected to ultracentrifugation for 2 h at 40,000 rpm using a SW41 Ti rotor (Beckman). Bands were collected from the top of each density interphase (F1, 0–10%; F2, 10–15%; F3, 15–20%) and analyzed by Western blot analysis.

##### BMP Synthesis in AML12 Cells

AML12 cells were cultured in DMEM/F-12 (1:1) containing 10% FCS, penicillin (100 units/ml), streptomycin (100 μg/ml), ITS (5 μg/ml insulin, 5 μg/ml transferrin, and 5 ng/ml selenium), and dexamethasone (40 ng/ml). BMP synthesis was determined by detecting the incorporation of ^3^H-labeled oleic into respective lipids ([^3^H]9,10-oleic acid, 2 μCi/well of a 6-well plate). For this purpose, confluent cells were cultivated for 24 h in DMEM/F-12 (1:1) containing 1% FCS. Subsequently, cells were exposed to 20% FCS containing ^3^H-labeled oleic acid for 4 h either in the absence or presence of KT182 (1 μm). Inhibitor or carrier (dimethyl sulfoxide) was added 1 h before exposure to 20% FCS/[^3^H]oleic acid. Total lipids were extracted twice with 1 ml hexan:isopropanol (3:2) and once with 1 ml isopropanol. Lipids were dried under nitrogen and redissolved in chloroform containing BMP standard. Subsequently, lipids were separated by TLC using chloroform:methanol:32% ammonia (65:35:5, v/v/v). After visualization by iodine staining, BMP and cholesteryl ester bands were cut out and subjected to scintillation counting.

##### LC-MS Analysis of BMP

Cells were extracted twice with 1 ml of hexan:isopropanol (3:2) containing 200 pmol/ml BMP 14:0 (Avanti polar lipids) as internal standard and once with 1 ml of isopropanol. The combined organic phases were evaporated under nitrogen and stored at −20 °C until further processing. Tissues were homogenized by an Ultra Turrax (IKA) in 0.8 ml of distilled water and extracted with 4 ml of chloroform/methanol 2:1 for 1 h by overhead shaking. To each sample, 400 pmol of BMP 14:0 was added. Samples were centrifuged (3000 rpm, 3 min) to induce phase separation, and the organic lower phase was collected. The aqueous phase was extracted once more with 2.7 ml of chloroform by overhead shaking for 15 min. After centrifugation (3000 rpm, 3 min), the organic phases were combined and evaporated under a stream of nitrogen. The anionic phospholipid fraction containing BMP was obtained by a solid-phase extraction of the lipid extract on self-made amino-functionalized silica gel cartridges (Sigma-Aldrich). In brief, the silica gel was washed with 4 ml of hexane before the samples were applied on the column in chloroform (2 × 0.5 ml). Then lipids were eluted with 4 ml of chloroform/methanol (2:1), 4 ml of diethylether containing 1% acetic acid, 4 ml of methanol, and 4 ml of chloroform/methanol/0.8 m sodium acetate 30/60/8 (v/v/v). The last fraction was collected in glass tubes and re-extracted with 3.67 ml of chloroform and 1.14 ml of distilled water. After phase separation by centrifugation (3000 rpm, 3 min, room temperature), the aqueous phase was discarded, and the organic phase was evaporated and reconstituted in 50 μl of 70/30 isopropanol/(chloroform/methanol, 2:1, v/v) for the LC-MS measurement. For quantification of BMP 36:2, a calibration curve was prepared ranging from 0.4–26 μm that was processed in the same way as the biological samples.

The LC-ESI-MS measurement was performed on an ACQUITY-UPLC system (Waters, Manchester, UK) coupled to a Synapt^TM^ G1 qTOF (Waters). The chromatographic separation was adapted from Knittelfelder *et al.* (22) using a HSS T3 vanguard and column (100 × 2.1 mm, 1.8 μm, Waters) and a gradient of 15 min of total run time with a flow rate of 0.3 ml/min. The following source parameters were used: capillary temperature, 100 °C; desolvatization temperature, 500 °C' N_2_ as nebulizer gas; capillary voltage (positive mode), 2.6 kV. The lock spray was achieved by an external pump (L-6200, Hitachi) at a flow rate of 0.5 ml/min split in a 1:13 ratio. 200 pg/μl leucine-enkephalin ((M + H)^+^, *m*/*z* 556.2771) was used as reference substance in the lock spray. Data acquisition was performed by MassLynx 4.1 software (Waters), and for lipid analysis the Lipid Data Analyzer software was used (23). The batch quantitation setup was as follows: retention time tolerance before/after, 0.1 min; relative base peak cutoff, 0.1‰; retention time shift, 0.1 min; and isotopic quantitation of two isotopes where one isotopic peak has to match.

##### Antisense Oligonucleotide-mediated Knockdown of ABHD6

For ABHD6 knockdown studies, male C57BL/6N mice (Harlan) at 6–8 weeks of age were either maintained on standard rodent chow or switched to a high-fat diet for a period for 12 weeks. Mice were injected biweekly with antisense oligonucleotides (ASOs) (25 mg/kg of body weight) targeting knockdown of ABHD6 exactly as described previously (14). The high-fat diet was prepared by the Wake Forest School of Medicine institutional diet core and contained 45% of energy as lard (16:0 = 23.3%, 18:0 = 15.9%, 18:1 = 34.8%, 18:2 = 18.7%). This diet has been described previously (14). The 20-mer phosphorothioate ASOs were designed to contain 20-0-methoxyethyl groups at positions 1–5 and 15–20 and were synthesized, screened, and purified as described previously (24) by ISIS Pharmaceuticals, Inc. (Carlsbad, CA).

##### Western Blot Analysis

Proteins of tissue homogenates and cell extracts were separated by 10% SDS-PAGE according to standard protocols and blotted onto a polyvinylidene fluoride membrane (Carl Roth GmbH). Endogenous ABHD6 expression was detected using a rabbit polyclonal antibody raised against murine ABHD6 (14). His-tagged proteins were detected using mouse anti-His antibody (GE Healthcare/Amersham Biosciences). Cytochrome oxidase subunit 4 (CoxIV), protein disulfide isomerase, lysosomal-associated membrane protein 1 (LAMP-1), and ras-associated protein 7 (RAB7A) were detected using rabbit anti-COXIV, anti-protein disulfide isomerase, anti-LAMP-1, and anti-rab7 antibodies (all from Cell Signaling Technology). After binding of the secondary anti-mouse or anti-rabbit HRP-linked antibodies, signals were visualized by enhanced chemiluminescence detection (ECL Plus/Prime, GE Healthcare).

##### Live-cell Imaging and Immunostaining

COS-7 cells were cultivated in 8-well coverslips (Sarstedt, Giesen, Germany) and co-transfected with ABHD6-ECFP, mRFP-Rab5, mRFP-Rab7protein disulfide isomerase (gifts from Ari Helenius (25)), or pDsRed2-ER (Clontech) using Metafectene (Biontex). 48 h after transfection, cells were imaged by confocal fluorescence microscopy using a Leica SP5 equipped with a Leica HCX 63 × 1.25 NA water immersion objective. Lipid droplet formation was induced by cultivating cells for 16 h in DMEM containing 400 μm oleic acid, and neutral lipids were stained using HCS LipidTOX^TM^ DeepRed (Invitrogen) according to the instructions of the manufacturer. ECFP was excited at 458 nm, and emission was detected between 470 and 500 nm. DsRed2 was excited at 514 nm, and emission was detected between 530 and 580 nm. mRFP was excited at 563 nm, and emission was detected between 580 and 630 nm. LipidTOX^TM^ DeepRed was excited at 633 nm, and emission was detected between 650 and 750 nm.

For immunostaining experiments, untagged ABHD6 was expressed in COS-7 cells as described above. 48 h after transfection, cells were fixed for 15 min in PBS containing 4% paraformaldehyde. Cells were permeabilized for 10 min with PBS containing 0.1% Triton X-100. Unspecific binding sites were blocked for 1 h at room temperature using 5% goat serum in PBS containing 0.1% Triton X-100. Subsequently, cells were incubated with primary antibody (rabbit Anti-ABHD6, 1:1000) overnight at 4 °C and secondary antibody (goat anti-rabbit Dylight 488, 1:4000, Thermo Scientific) for 1 h at room temperature. Every incubation step was followed by three washing steps at room temperature for 10 min using PBT. Cells were mounted using Vectashield® (Vector Laboratories) mounting medium and imaged using a Leica SP5 as described above.

##### Statistical Analysis

Data are shown as mean ± S.D. Statistical significance was determined by Student's unpaired *t* test (two-tailed) or one-way analysis of variance followed by Dunnett's post hoc test. Group differences were considered statistically different at *p* < 0.05 (*), *p* < 0.01 (**), and *p* < 0.001 (***).

## Results

### 

#### 

##### ABHD6 Degrades BMP

**FIGURE 1.**
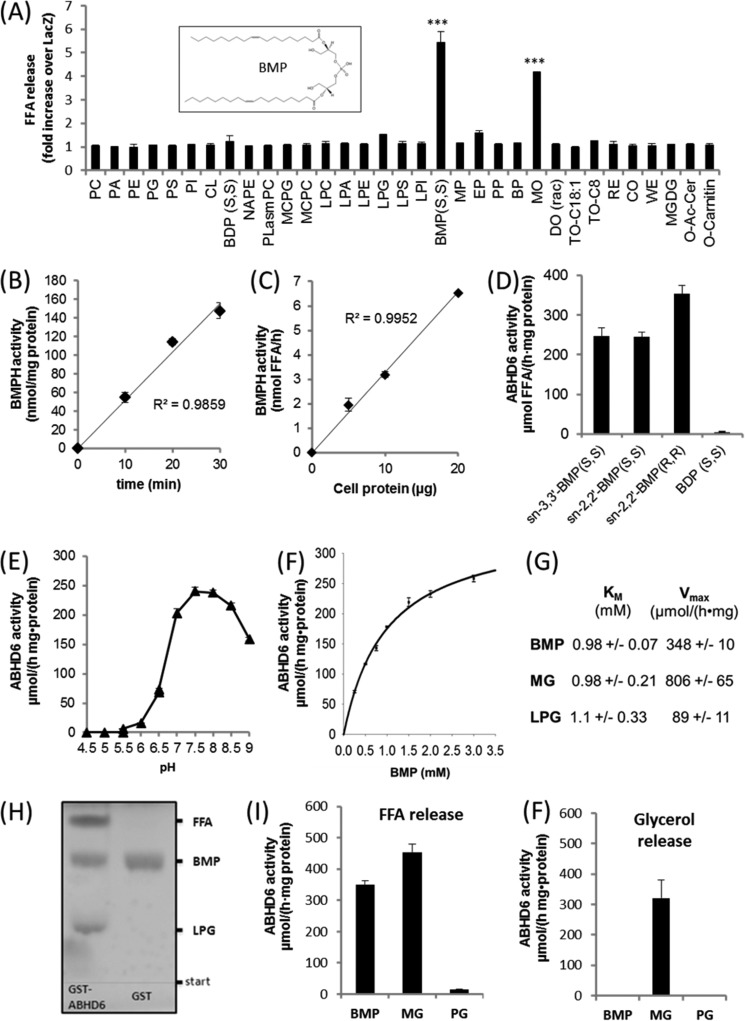
**Substrate selectivity and pH dependence of ABHD6.** *A*, the coding sequence of mouse ABHD6 was cloned into a mammalian expression vector (pcDNA4/HisMaxC) and overexpressed in COS-7 cells. Cell lysates (1000 × *g* supernatant) containing recombinant ABHD6 were incubated with various polar and neutral lipids. Lipid degradation was monitored by measuring the release of FFA. Cells expressing β-galactosidase (LacZ) were used as a negative control. Data are expressed as -fold increase in FFA release over LacZ. For more information about lipids, see “Experimental Procedures.” *Inset*, *sn-*2,2′-dioleoyl BMP. *B* and *C*, time- (*B*) and dose-dependent (*C*) hydrolysis of BMP by Cos-7 lysates overexpressing ABHD6. The activity detected in cells expressing LacZ was set as blank. *D*, degradation of *sn-*3,3′-BMP(*S*,*S*), *sn-*2,2′-BMP(*S*,*S*), *sn-*3,3′-BMP (*R*,*R*), and bis(diacylglycero)phosphate(*S*,*S*) by purified GST-tagged ABHD6. *E*, pH dependence of purified GST-ABHD6 using BMP(*S*,*S*) as substrate. Activity assays were performed in 50 mm acetate (pH 4.5–5.5), MES (pH 5.5–6.5), and bis-tris propane buffer (pH 6.5–9.0). *F*, substrate saturation kinetics of purified GST-ABHD6 using BMP(*S*,*S*) as substrate. *G*, *K_m_* and *V*_max_ values using BMP(*S*,*S*), racemic MO, and LPG as substrate. Values were calculated by nonlinear regression analysis (SigmaPlot). *H*, TLC analysis showing that partial digestion of BMP with GST-ABHD6 results in the accumulation of LPG and FFA. After digestion, lipids were isolated by butanolic extraction and applied to TLC analysis using chloroform/methanol/water (60/25/4, v/v/v) as solvent. GST purified under identical conditions served as a negative control. *I* and *F*, FFA (*I*) and glycerol (*F*) release using BMP(*S*,*S*), racemic MO, or PG as substrate for GST-ABHD6. FFA and glycerol release were quantified using commercially available kits. Data are presented as mean ± S.D. (***, *p* < 0.001, Student's *t* test).

We have shown previously that purified ABHD6 degrades MG and acidic lysoglycerophospholipids with a preference for lysophosphatidylglycerol (LPG) (14). To further characterize the enzymatic function of ABHD6, we overexpressed ABHD6 in COS-7 cells, incubated cell lysates with a variety of ester bond-containing lipid substrates, and determined the release of FFA in comparison with control cells transfected with β-galactosidase (LacZ). We observed a severalfold increase in FFA release using BMP and racemic-monoolein (MO) as substrate. In comparison, low activity was observed for LPG and ethylpalmitate. These observations indicate that BMP and MG are the preferred substrates of ABHD6 (Fig. 1*A*). Further experiments revealed that overexpression of ABHD6 increases the BMPH activity of cell lysates in a dose- and time-dependent manner (Fig. 1, *B* and *C*).

Next, we expressed GST-tagged murine ABHD6 in *S. cerevisiae*, purified the GST-tagged enzyme by affinity chromatography as described previously (14), and performed activity assays using BMP as substrate. Naturally occurring BMP has an unusual *sn*-1,1′diglycerophosphate backbone (BMP(*S*,*S*)), and the fatty acids are rather bound in the *sn-2* than in the *sn-3* position of the glycerol moieties (Fig. 1*A*, *inset*, shows *sn-*2,2′-dioleoyl BMP) (17). ABHD6 exhibited almost identical activities using *sn-*3,3′- or *sn-*2,2′-dioleoyl BMP (both *S*,*S*) as substrate (Fig. 1*D*). *sn-*3,3′-Dioleoyl BMP (*R,R*), having a *sn*-3,3′ diglycerophosphate backbone, was degraded with somewhat higher activity than the *S*,*S* isoforms, whereas bis(diacylglycero)phosphate was not hydrolyzed (Fig. 1*D*). The specific activity of different purified ABHD6 preparations varied between ∼100 and 400 μmol/(h·mg of protein) (equal to 1.7–6.7 units/mg), suggesting that ABHD6 is capable of degrading BMP with high efficiency (data not shown). Together, these observations implicate that the enzyme can act as BMPH, has no positional preference, and does not distinguish between stereoisomers of BMP.

ABHD6 exhibited a pH optimum of between 7.5 and 8.0 (Fig. 1*E*) and lacked activity under acidic conditions. Substrate saturation measurements revealed a *K_m_* of ∼1 mm. (Fig. 1*F*). Under the same conditions, similar *K_m_* values were obtained using MO and LPG as substrates (Fig. 1*G*), suggesting that ABHD6 exhibits comparable affinities for these substrates. However, calculated *V*_max_ values for LPG were 9- and 3.9-fold below that obtained for MG and BMP, respectively, which suggests that LPG is hydrolyzed with lower velocity (Fig. 1*G*). In accordance with this observation, we observed that ABHD6-mediated hydrolysis of BMP leads to accumulation of the intermediary product LPG (Fig. 1*H*). Furthermore, ABHD6-mediated hydrolysis of MO generated FFA and glycerol (Fig. 1*I*/F), whereas degradation of BMP produced FFA but not glycerol. This excludes that ABHD6 mobilizes MG from the BMP molecule, which would be degraded further into FFA and glycerol by the enzyme. PG, the structural isomer of BMP, was not hydrolyzed.

##### ABHD6 Acts as a Late Endosome/Lysosome-associated Lipid Hydrolase

**FIGURE 2.**
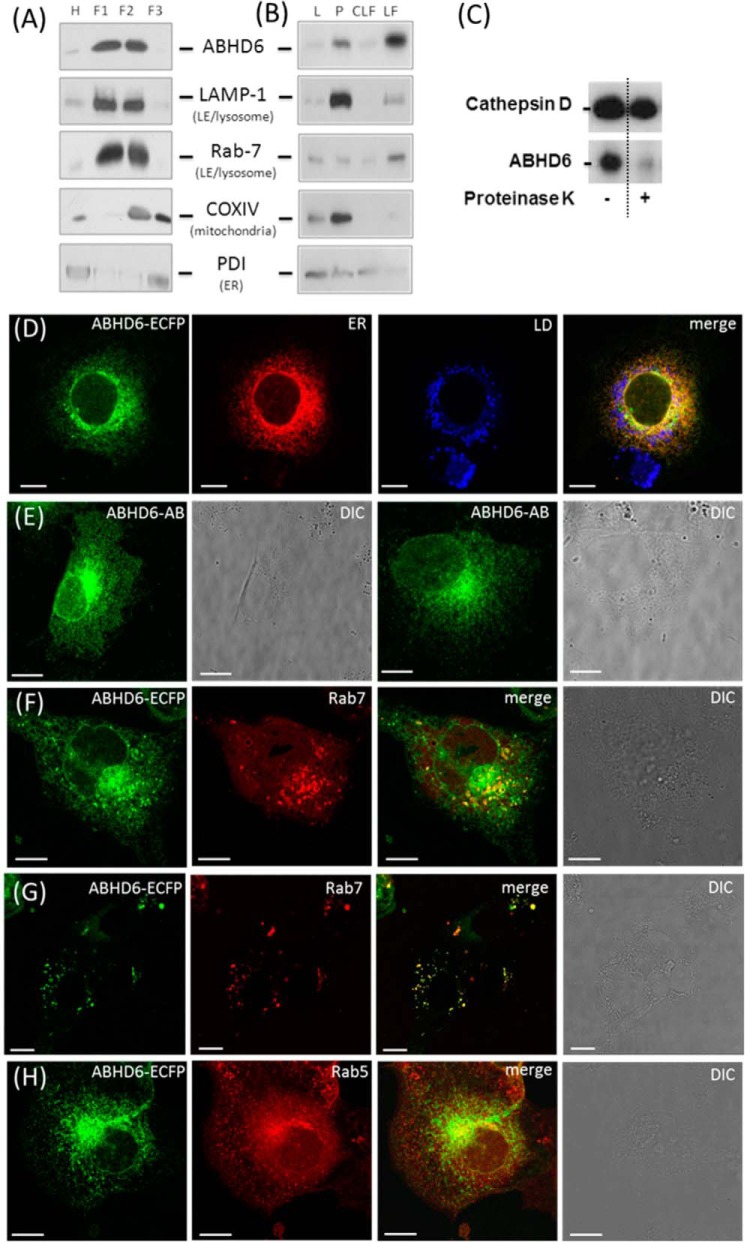
**ABHD6 co-localizes with late endosomes/lysosomes.** *A* and *B*, lysosomal fractions of liver homogenates from overnight-fasted C57Bl/6 animals were isolated by (*A*) ultracentrifugation in a discontinuous OptiPrep™ gradient or (*B*) differential centrifugation. 10 μg of protein of various fractions was analyzed by Western blotting using an antibody specific for ABHD6 (14) and marker antibodies for LE/lysosomes (Rab-7 and LAMP-1), ER (PDI, protein disulfide isomerase), and mitochondria (COXIV). *L*, lysate (1000 × *g* supernatant); *F1-F3*, fractions 1–3 harvested from the top of the gradient at the interphase 0–10%, 10–15%, and 15–20% iodixanol, respectively; *P*, 12,000 × *g* pellet; *CLF*, crude lysosomal fraction; *H*, homogenate. *C*, protease protection assay performed with lysosomal liver fractions obtained using OptiPrep^TM^ gradient (F1). The F1 fraction was treated with 30 ng pf proteinase K at 37 °C for 5 min or carrier alone (control). Subsequently, proteins were precipitated with acetone and subjected to Western blot analysis. Proteins were detected using specific primary antibodies against Cathepsin D (Abcam) and ABHD6. *D*, live-cell imaging of COS-7 cells transfected with ABHD6-ECFP and co-stained with ER-Tracker DsRED2 (*ER*) and lipid droplet (*LD*) marker (LipidTOX^TM^ DeepRed, Invitrogen). Also shown is immunofluorescence of COS-7 transfected with tagless ABHD6. The enzyme was detected using ABHD6-specific antibody and a fluorescent labeled secondary antibody. Non-transfected cells were not stained by the antibody and served as a negative control. *E* and *F*, live-cell imaging of COS-7 cells co-transfected with ABHD6-ECFP and the LE marker RFP-Rab7. *DIC*, differential interference contrast. *G*, live-cell imaging of COS-7 cells co-transfected with ABHD6-CFP and the early endosome marker RFP-Rab5. (*Scale bar* = 10 μm).

Because BMP is enriched in LE and lysosomes, we hypothesized that ABHD6 must also localize to these organelles when acting as BMPH. To get a first insight into the subcellular localization of the enzyme, liver lysates from overnight-fasted mice were fractionated by ultracentrifugation in a discontinuous OptiPrep^TM^ gradient. Aliquots of the fractions were analyzed by Western blotting using a polyclonal ABHD6-specific antibody and marker proteins specific for LE/lysosomes (Rab7/Lamp-1), mitochondria (CoxIV), and ER (protein disulfide isomerase). ABHD6 was detected in density fractions containing Lamp-1 and Rab7 (F1 and F2) but not in the F3 fraction enriched in mitochondria (Fig. 2*A*). Similar data were obtained when homogenates were subjected to differential centrifugation into a 1000 × *g* supernatant (L), a 12,000 × *g* pellet (P), a crude lysosomal fraction (12,000 × *g* supernatant), and an LF (25,000 × *g* pellet). ABHD6 was enriched in the 12,000 × *g* pellet compared with the 1000 × *g* supernatant. However, further purification of the crude lysosomal fraction clearly revealed further enrichment of ABHD6 in the LF together with Lamp-1 and Rab7 (Fig. 2*B*). To exclude that ABHD6 is imported in LE/lysosomes, we performed a protease protection assay. For that purpose, we digested proteins of the F1 fraction (Fig. 2*A*) with proteinase K and monitored the degradation of ABHD6 and cathepsin D, a luminal lysosomal protein that should be protected from degradation by the outer membrane of lysosomes. As shown in Fig. 2*C*, ABHD6, but not cathepsin D, was degraded by proteinase K treatment, indicating that ABHD6 is not a luminal protein.

To confirm the subcellular localization of ABHD6, we performed live-cell imaging experiments. COS-7 cells overexpressing fluorescent protein-tagged ABHD6 (ABHD6-ECFP) were analyzed by confocal laser-scanning microscopy. ABHD6-ECFP emerged enriched in ER membrane structures, concentrated in the perinuclear area, and did not show the typical ring-shaped lipid droplet localization, suggesting that it is not a lipid droplet-associated protein (Fig. 2*D*). The perinuclear localization pattern was also observed when ABHD6 was expressed without a tag and detected using an ABHD6-specific antibody (*ABHD6-AB*, Fig. 2*E*) indicating that the tag does not affect subcellular localization. Importantly, ABHD6 appeared on vesicles positive for Rab7 (Fig. 2*F*), which is typically found on LE and lysosomes (26). In cells exhibiting lower transgene expression, the co-localization of ABHD6-ECFP and Rab7 was more prominent (Fig. 2*G*). In contrast, ABHD6 did not co-localize with peripheral Rab5-positive early endosomes (Fig. 2*H*). Therefore, tissue fractionation and live-cell imaging experiments suggest that ABHD6 co-localizes with LE/lysosomes.

##### BMP Is Resistant to Acid Hydrolysis and Degraded Efficiently in the Slightly Alkaline pH Range in a Process Involving ABHD6

**FIGURE 3.**
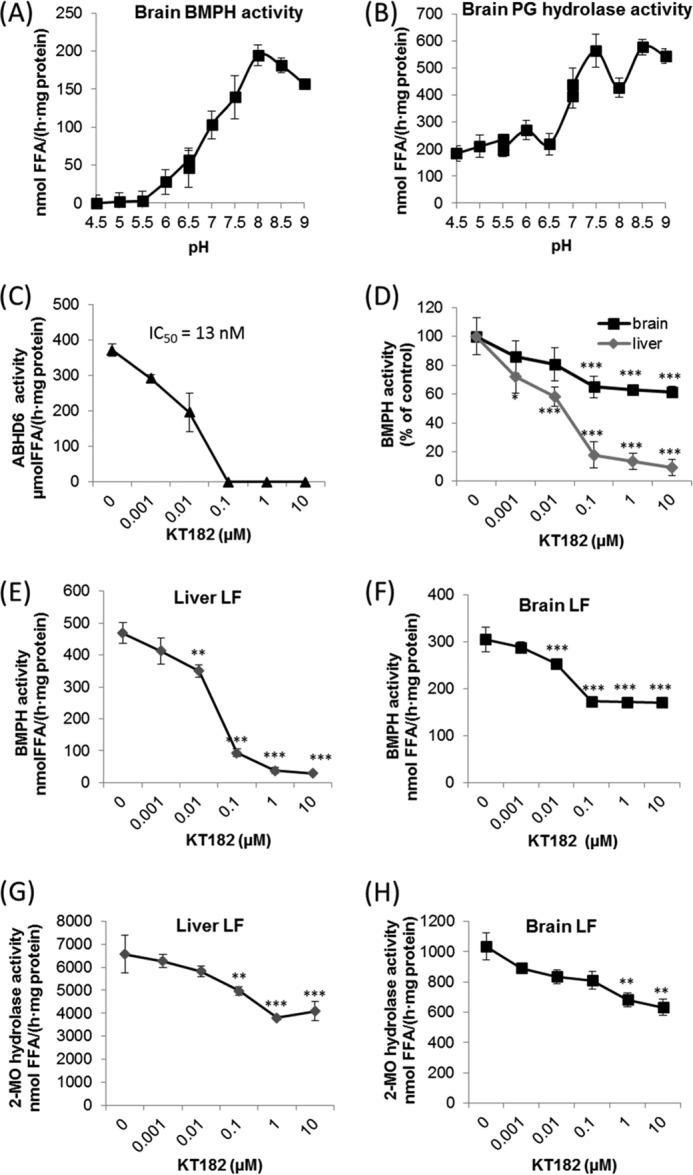
**BMP is resistant to lysosomal hydrolases and degraded efficiently in the slightly alkaline pH range in a process involving ABHD6.** *A* and *B*, pH dependence of (*A*) BMP and (*B*) PG hydrolase activity of brain lysates. Degradation of BMP(*S*,*S*) and PG (2 mm each) was monitored between pH 4.5 and 9.0 as described in Fig. 1*D. C*, inhibition of BMPH activity of purified ABHD6 by KT182 using BMP(*S*,*S*) as substrate. *D*, inhibition of brain and liver BMPH activity by KT182. *E* and *F*, inhibition of BMPH activity by KT182 in LF of liver and brain. LF were purified as described in Fig. 2*B. G* and *H*, inhibition of MG hydrolase activity in LF of liver and brain by KT182. *C—H*, activity assays were performed at pH 8.0 using BMP(*S*,*S*) and 2-monoolein (*2-MO*) as substrate (2 mm each). Data are presented as mean ± S.D. and represent at least three independent experiments. *, *p* < 0.05; **, *p* < 0.01; ***, *p* < 0.001; analysis of variance.

To compare the characteristics of purified ABHD6 with tissue activity, we used brain lysates as a source for BMPH activity. Interestingly, brain lysates did not contain detectable BMPH activity at acidic pH, and the most efficient BMP degradation occurred at pH 8 (Fig. 3*A*), similar to what was observed for purified ABHD6 (Fig. 1*E*). Conversely, the structural isomer PG was degraded in the acidic and in the alkaline range (Fig. 3*B*). The resistance of BMP to acid hydrolysis and its efficient hydrolysis under mildly alkaline conditions indicate that degradation of BMP may occur in non-acidic compartments, most likely on the outer membrane of LE/lysosomes.

To investigate the contribution of ABHD6 to brain and liver BMPH activity at pH 8.0, we used the ABHD6-specific inhibitor KT182. In agreement with published data (27), this compound inhibited purified ABHD6 with an IC_50_ value of ∼13 nm (Fig. 3*C*). In brain lysates, KT182 inhibited BMPH activity in a dose-dependent manner up to 40% (Fig. 3*D*). Notably, liver BMPH activity was reduced by more than 90%, suggesting a major role of ABHD6 in hepatic BMP catabolism (Fig. 3*D*).

Next, we investigated the contribution of ABHD6 to the BMPH activity associated with the lysosomal fraction of liver and brain isolated by differential centrifugation (Fig. 2*B*). The ABHD6 inhibitor KT182 almost completely abolished BMPH activity in hepatic LF fractions (Fig. 3*E*). In LF of brain, KT182 reduced BMPH activity by ∼50% (Fig. 3*F*), again indicating tissue-specific differences in BMP catabolism. Because ABHD6 has been reported to degrade MG, we determined MG hydrolase activity associated with lysosomes using 2-monoolein as substrate. KT182 reduced MG hydrolase activity up to ∼40% in both liver and brain preparations (Fig. 3, *G* and *H*). These observations imply that ABHD6 acts as an LE/lysosome-associated BMP and MG hydrolase. The stronger effect of KT182 on BMPH activity suggests that ABHD6 influences BMP rather than MG catabolism.

##### ABHD6 Affects BMP Catabolism in Vitro and in Vivo

**FIGURE 4.**
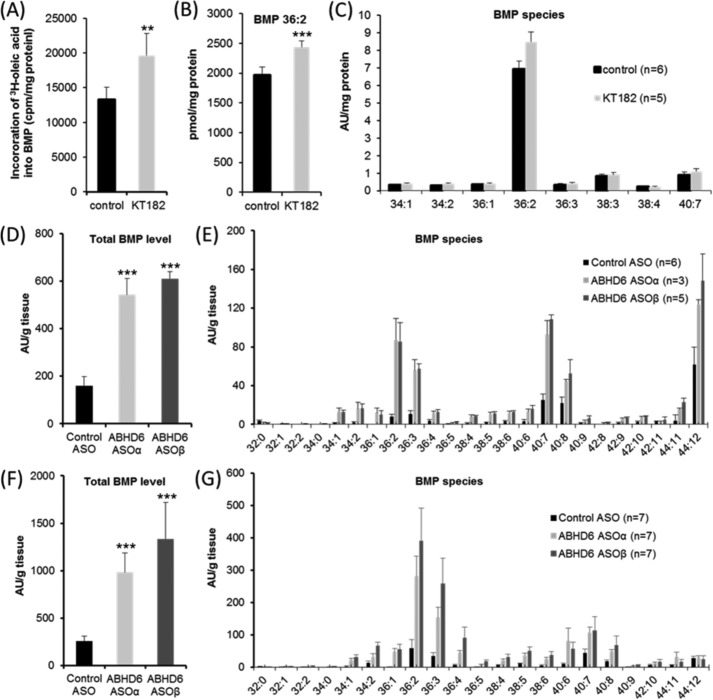
**ABHD6 affects BMP catabolism.** *A*, incorporation of ^3^H-labeled oleic acid into BMP of AML12 hepatocytes. Cells were cultivated for 24 h in DMEM/F-12 (1:1) containing 1% FCS. Subsequently, cells were exposed to DMEM/F-12 (1:1) containing 20% FCS and ^3^H-labeled oleic acid for 4 h either in the absence or presence of KT182. Inhibitor or carrier (dimethyl sulfoxide) was added 1 h before exposure to DMEM/F-12 (1:1) containing 20% FCS/[^3^H]oleic acid. Total lipids were extracted and separated by TLC analysis. The radioactivity co-migrating with BMP was determined by scintillation counting. *B* and *C*, effect of KT182 on BMP level and distribution in AML12 cells. Cells were cultivated under identical conditions as described in *A* but in the absence of ^3^H-labeled oleic acid. Total lipids were extracted in the presence of BMP 14:0 as internal standard and subjected to LC-MS analysis. For quantification of BMP 36:2 (*B*), a calibration curve was prepared. Other molecular species are shown in arbitrary units (*AU*)/mg of protein. *D–G*, liver BMP levels of male C57BL/6N mice treated with ASOs targeting ABHD6 (ASOα and ASOβ) or control ASO. Mice were maintained for 12 weeks on a normal chow diet or on a high-fat diet and injected biweekly with ASOs (25 mg/kg of body weight) as described previously (14). Subsequently, lipids were extracted from liver samples in the presence of BMP 14:0 (Avanti Polar Lipids) as internal standard and subjected to LC-MS analysis. *D* and *E*, relative total BMP levels (*D*) and BMP distribution (*E*) of mice maintained on a chow diet. *F* and *G*, relative total BMP levels (*F*) and BMP distribution (*G*) of mice maintained on a high-fat diet. The experiments shown in *A–C* are representative of two independent experiments performed in triplicate. Data are presented as mean ± S.D. *, *p* < 0.05; **, *p* < 0.01; ***, *p* < 0.001; analysis of variance).

To examine the effect of ABHD6 inhibition on cellular BMP metabolism, we first monitored the incorporation of radiolabeled oleic acid into BMP of AML12 hepatocytes in the presence and absence of KT182. Western blot analysis revealed that these cells express ABHD6 (data not shown), and pharmacological inhibition reduced BMPH activity in AML12 lysates by 41% ± 5% (*p* < 0.01). To monitor BMP synthesis, cells were moderately starved for 24 h in DMEM:F12 (1:1) medium containing 1% FCS and then exposed to the same medium containing 20% FCS and [^3^H]oleic acid for 4 h. Under these conditions, KT182 increased [^3^H]oleic acid incorporation into BMP by 48% in comparison with control cells, indicating that ABHD6 inhibition slows down BMP degradation (Fig. 4*A*). To confirm this observation, we determined cellular BMP levels by LC-MS under identical conditions. Dioleoyl-BMP, the major BMP species in AML12 cells, was increased by 24% in inhibitor-treated cells (Fig. 4*B*), whereas other molecular species were not affected (Fig. 4*C*). Therefore, ABHD6 inhibition in AML12 cells causes a moderate increase in BMP levels under the applied conditions.

The *in vivo* effect of ABHD6 on BMP catabolism was investigated using two ABHD6 antisense oligonucleotides (ASOα and ASOβ), leading to almost complete knockdown of ABHD6 in liver, as described previously (14). In mice fed a normal chow diet, ABHD6 knockdown increased total hepatic BMP levels more that 3-fold in comparison with mice treated with control ASO (Fig. 4*D*). We detected MS signals for 24 different BMP species, and the majority of these species were increased in response to ASOα as well as ASOβ treatment (Fig. 4*E*). Similar results were obtained in mice fed a high-fat diet. In comparison with control ASO, ASOα and ASOβ increased total BMP levels 3.8- and 5.2-fold, respectively (Fig. 4*F*). These data demonstrate that ABHD6 affects hepatic BMP catabolism *in vivo*. Additionally, we observed that mice receiving the high-fat diet exhibit higher total BMP levels than chow diet-fed mice (1.6-, 1.8-, and 2.2-fold in control ASO, ASOα, and ASOβ-treated mice, respectively) and that the high-fat diet strongly changes the relative composition of BMP species (Fig. 4, compare *D* and *F* with *E* and *G*). Most obvious was the high-fat diet-induced relative decrease in BMP esterified with docosahexaenoic acid (22:6), which occurred independent of ABHD6 knockdown (Fig. 4*G*).

##### Identification of Mutations of Human ABHD6 Lacking BMPH Activity

**FIGURE 5.**
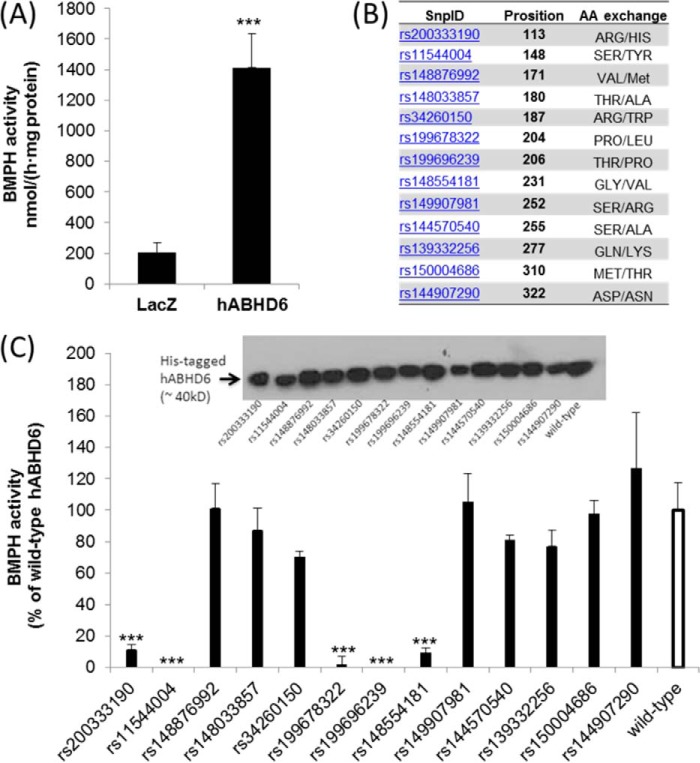
**Activity of human ABHD6 and mutant variants of the enzyme.** *A*, the coding sequence of hABHD6 was cloned into a mammalian expression vector (pcDNA4/HisMaxC), and the protein was expressed in COS-7 cells. Mutations were introduced by site-directed mutagenesis. For enzymatic assays, cell lysates (1000 × *g* supernatant) were incubated with BMP(*S*,*S*), and FFA release was determined. Lysates obtained from cells expressing β-galactosidase (LacZ) were used as a negative control. *B*, selected mutations of hABHD6 as reported by the NCBI dbSNP database (http://www.ncbi.nlm.nih.gov/projects/SNP). *C*, BMPH activity of wild-type hABHD6 and mutant variants of the enzyme. Data are expressed as percent activity of wild-type hABHD6 and were normalized to the expression levels of His-tagged proteins. The activity detected in lysates of LacZ-expressing cells was set as blank. *Inset*, Western blot of His-tagged variants using an anti-His antibody. Data are presented as mean ± S.D. ***, *p* < 0.001; analysis of variance.

Both mouse and human ABHD6 (hABHD6) exhibit MG hydrolase activity (28). To test whether hABHD6 also exhibits BMPH activity, we overexpressed His-tagged proteins in COS-7 cells. In comparison with control cells expressing LacZ, ABHD6 transfection increased cellular BMPH activity 7-fold (Fig. 5*A*). Several SNPs have been reported for this protein, leading to the exchange of single amino acids (http://www.ncbi.nlm.nih.gov/projects/SNP, Fig. 5*B*), whereby the mutation at Ser-148 (rs11544004) affects the predicted active serine within the conserved G*X*S*X*G motif of α/β hydrolases (28). Some of these missense variants were cloned by site-directed mutagenesis and expressed in COS-7 cells. rs11544004 as well as two other point mutations (rs199678322 and rs199696239) led to complete loss of BMPH activity. rs200333190 and rs148554181 exhibited strongly reduced activity in comparison with the wild-type enzyme (Fig. 5*C*). Therefore, both human and mouse ABHD6 are capable of degrading BMP, and it is likely that loss-of-function mutations are present in humans.

## Discussion

ABHD6 is thought to contribute to MG hydrolysis in cells lacking monoglyceride lipase, such as pancreatic β cells and specific cell types of the brain (5, 15). Our data demonstrate that ABHD6 has an additional function. The enzyme affects BMP catabolism and is therefore part of the LE/lysosomal cargo-sorting machinery. BMP is present in small amounts in most tissues and highly enriched in ILVs of LE, where it represents ∼15% of total phospholipids (16). Because of its cone-shaped structure, BMP can induce membrane asymmetry, favoring the formation of ILVs in LE (29). BMP-containing ILVs play an important role in lipid digestion and sorting. BMP is negatively charged at lysosomal pH and highly resistant to degradation in acidic compartments. Because of these features, it is believed that BMP can form a stable docking station for luminal acid hydrolases and lipid-binding proteins that are positively charged at acidic pH (18). The presence of luminal membrane structures is specifically important for lipases because these enzymes require a water-lipid interphase for activation. Accordingly, it has been shown that BMP stimulates the activity of a number of lysosomal lipid-degrading enzymes, including acid sphingomyelinase (30), acid ceramidase (31), acid phospholipase A_2_ (32), and lysosomal acid lipase (33). The latter enzyme is responsible for triglyceride and cholesterylester degradation in lysosomes. Furthermore, BMP is thought to play an important role in endosomal cholesterol sorting. BMP accumulation has been reported in Niemann-Pick disease, type C (NPC) and various other genetic forms of lysosomal storage disease (17). NPC is caused by mutations in the NPC1 or NPC2 gene, leading to accumulation of cholesterol and glycosphingolipids in LE/lysosomes (34). Currently it is unknown whether BMP accumulation is a secondary event because of a traffic jam in acidic organelles or whether it contributes to the pathogenesis of lysosomal storage disease (17). However, because BMP acts as activator of lipid digestion and sorting, it can be assumed that BMP counteracts the pathological accumulation of lipids in LE/lysosomes. Accordingly, published data suggest that exogenously added BMP can improve the phenotype of NPC fibroblasts. Blocking BMP function using an anti-BMP antibody increases the cellular cholesterol content and alters cholesterol distribution (17, 35, 36). Furthermore, the NPC2-mediated transfer of cholesterol between membranes is increased drastically by the presence of BMP in donor vesicles (37). In addition to genetic forms of lysosomal storage diseases, BMP levels have been shown to increase in drug-induced lipidosis, which can be caused by cationic amphiphilic drugs such as amiodarone (38). Such compounds bind to the negatively charged BMP in lysosomes and, consequently, block lipid degradation, leading to the accumulation of undigested material (17). BMP is also present in serum and a clinical biomarker for drug-induced lipidosis. This implies that the lipid is secreted under pathophysiological conditions (39), most likely via exosomes (40).

Despite its important role in endosome maturation and lipid sorting, the anabolic and catabolic pathways of BMP metabolism are incompletely understood (17). Our observations indicate that cells/tissues lack acid BMPH activity, which is a prerequisite for forming stable membrane structures in acidic compartments. Tissue BMPH activity is highest at a slightly alkaline pH range, indicating that the lipid is degraded efficiently by enzymes that are active under cytosolic conditions. We show that ABHD6 is responsible for ∼90% and 50% of the BMPH activity detected in liver and brain, respectively, indicating tissue-specific differences in BMP catabolism. ABHD6 knockdown drastically increased hepatic BMP levels, demonstrating an important role of the enzyme in BMP degradation *in vivo*. In comparison, pharmacological inhibition of ABHD6 in AML12 cells produced a moderate increase in BMP levels, which might be due to low turnover of BMP in these cells. It is also interesting to note that BMP levels and composition changed in response to high-fat feeding. To our knowledge, this has not been reported before. However, this observation is not surprising considering that lysosomes play an active role in the breakdown of endocytosed material, autophagy, and hepatic energy stores and that autophagy malfunction is thought to contribute to the development of fatty liver disease (41).

**FIGURE 6.**
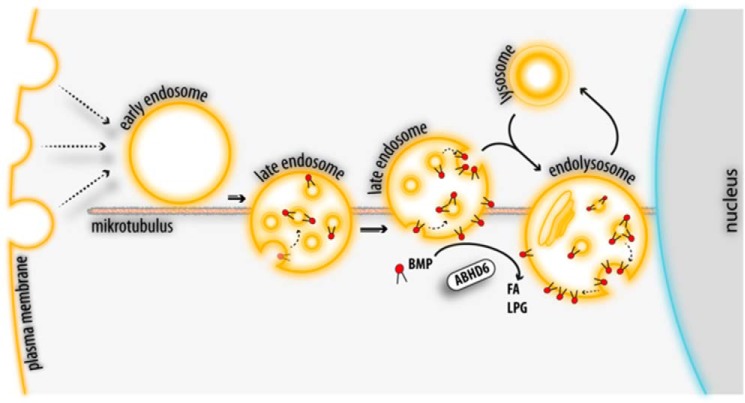
**Proposed role of ABHD6 in the late endosomal/lysosomal pathway.** Endocytic vesicles form early endosomes in the peripheral cytoplasm that move toward the perinuclear area along microtubuli. Their conversion into LE involves the formation of BMP-containing ILVs. LE finally fuse with lysosomes to form endolysosomes (51). BMP is resistant to acid hydrolysis and forms stable membrane structures within acidic organelles, promoting lipid digestion and sorting. BMP-rich ILVs of LE and endolysosomes can fuse with the limiting membrane, allowing the export of ILV contents. After back-fusion, BMP appears on the limiting membrane and is degraded by ABHD6 into LPG and FFA. Additionally, ABHD6 may counteract *de novo* BMP synthesis on the limiting membrane of LE.

BMP is predominantly found on luminal vesicles of acidic organelles and, consequently, not accessible for cytosolic enzymes. Therefore, the question arises of how ABHD6 gets access to BMP. Tissue fractionation and live-cell imaging experiments revealed that ABHD6 co-localizes with LE/lysosomes, suggesting that it can act on lipids present on the outer membrane. Importantly, ILVs can fuse with the limiting membrane of acidic organelles, and BMP may transiently become part of the limiting membrane (42). Back-fusion of ILVs is enhanced by BMP and the ESCRT accessory protein Alix and can represent an export route for endosomal cargo (19, 42). For example, this back-fusion process favors the fusion of endocytosed viruses, such as vesicular stomatitis virus (43) and human immunodeficiency virus (44), with the outer membrane of LE, allowing the release of their replication machinery into the cytosol. Furthermore, BMP has been shown to promote the biogenesis of ILVs deriving from the limiting membrane (29), which also implicates that BMP is transiently present on the outer membrane of LE. Accordingly, we hypothesize that ABHD6 degrades BMP after back-fusion of ILVs with the limiting membrane and/or limits *de novo* BMP synthesis (summarized in Fig. 6).

ABHD6 hydrolyzes BMP with high specific activity, but it is clearly not the only enzyme capable of catalyzing this reaction. Published work suggests that a member of the carboxylesterase family (45), pancreatic lipase related protein 2 (PLRP2) (46), and ABHD12 (47) can degrade BMP *in vitro*. PLRP2 is found in the pancreatic juice but might also possess intracellular functions. The enzyme hydrolyzes triglycerides, phospholipids, and galactolipids and is involved in dietary fat absorption and T cell-mediated cytotoxicity (48). However, the *in vivo* role of PLRP2 in BMP metabolism is unknown. Similar to ABHD6, ABHD12 was originally described as a 2-AG-degrading enzyme (1), and mutations of ABHD12 cause the rare inherited disorder polyneuropathy, hearing loss, ataxia, retinitis pigmentosa, and early-onset cataract (PHARC) (49). ABHD12 has been identified recently as an important lysophosphatidylserine lipase in mouse brain (47). This enzyme may substantially contribute to BMP hydrolysis in brain because mice lacking ABHD12 exhibit an ∼50% reduction in brain BMPH activity at pH 7.5 (47). ABHD12 is highly expressed in the brain and barely present in the liver (50). This tissue distribution could explain the high ABHD6-independent BMPH activity detected in brain but not in liver lysates.

In conclusion, our data show that ABHD6 affects BMP homeostasis and localizes to LE/lysosomes. Considering the central role of BMP in cargo sorting and ILV formation, it is reasonable to assume that ABHD6 exerts complex effects on the function of LE/lysosomes. It remains to be investigated whether changes in lipid sorting or cargo recycling can contribute to cannabimimetic (5) and anti-steatotic effects (14) provoked by ABHD6 inactivation.

## Author Contributions

R. Z. and J. M. B. designed the study and wrote the paper. M. A. P., I. M., G. F. G., U. T., B. S., C. H., M. R., A. L., H. W., L. G., J. K., and G. M. performed cell biological and biochemical experiments. O. K. and T. O. E. performed lipid analyses. S. H., F. A., and R. B. synthesized lipid standards. All authors reviewed and approved the final version of the manuscript.

## Footnotes

3 The abbreviations used are:

MG: monoacylglycerol
2-AG: 2-arachidonoylglycerol; bis(monoacylglycero)phosphate
ILV: intraluminal vesicle
LE: late endosome(s)
PG: phosphatidylglycerol
BMPH: bis(monoacylglycero)phosphate hydrolase
LF: lysosomal fraction
FFA: free fatty acid(s)
ASO: antisense oligonucleotide
LPG: lysophosphatidylglycerol
MO: monoolein
ER: endoplasmic reticulum
hABHD6: human ABHD6
NPC: Niemann-Pick disease, type C
ESI: electrospray ionization.
